# Dr. J. C. D.

**Published:** 1875-08

**Authors:** 


*Dr. J. C. D.*

We agree with our Tennessee friend in the opinion "that a medical journal falls short in the accomplishment of its full design without a good advertising department." We promise in future to pay more attention to that department. We shall not only request our readers to see that those whom we can endorse are patronized by their local druggist, but to warn them against.

